# Advertising, supplements and the role of the correspondence column

**DOI:** 10.1590/S1516-31802000000100007

**Published:** 2000-01-02

**Authors:** International Committee of Medical Journal Editors

## ADVERTISING

Most medical journals carry advertising, which generates income for their publishers, but advertising must not be allowed to influence editorial decisions. Editors must have full responsibility for advertising policy. Readers should be able to distinguish readily between advertising and editorial material. The juxtaposition of editorial and advertising material on the same products or subjects should be avoided, and advertising should not be sold on the condition that it will appear in the same issue as a particular article.

Journals should not be dominated by advertising, but editors should be careful about publishing advertisements from only one or two advertisers as readers may perceive that the editor has been influenced by these advertisers.

Journals should not carry advertisements for products that have proved to be seriously harmful to health—for example, tobacco. Editors should ensure that existing standards for advertisements are enforced or develop their own standards. Finally, editors should consider all criticisms of advertisements for publication.

## SUPPLEMENTS

Supplements are collections of papers that deal with related issues or topics, are published as a separate issue of the journal or as a second part of a regular issue, and are usually funded by sources other than the journal's publisher. Supplements can serve useful purposes: education, exchange of research information, ease of access to focused content, and improved cooperation between academic and corporate entities. Because of the funding sources, the content of supplements can reflect biases in choice of topics and viewpoints. Editors should therefore consider the following principles.

1. The journal editor must take full responsibility for the policies, practices, and content of supplements. The journal editor must approve the appointment of any editor of the supplement and retain the authority to reject papers.
2. The sources of funding for the research, meeting, and publication should be clearly stated and prominently located in the supplement, preferably on each page. Whenever possible, funding should come from more than one sponsor.
3. Advertising in supplements should follow the same policies as those of the rest of the journal.
4. Editors should enable readers to distinguish readily between ordinary editorial pages and supplement pages.
5. Editing by the funding organization should not be permitted.
6. Journal editors and supplement editors should not accept personal favors or excessive compensation from sponsors of supplements.
7. Secondary publication in supplements should be clearly identified by the citation of the original paper. Redundant publication should be avoided.

## THE ROLE OF THE CORRESPONDENCE COLUMN

All biomedical journals should have a section carrying comments, questions, or criticisms about articles they have published and where the original authors can respond. Usually, but not necessarily, this may take the form of a correspondence column. The lack of such a section denies readers the possibility of responding to articles in the same journal that published the original work.

